# Orofacial antinociceptive activity of codeine-associated geraniol in mice: a controlled triple-blind study

**DOI:** 10.1590/1807-3107bor-2024.vol38.0071

**Published:** 2024-08-05

**Authors:** Ana Paula Lopes NUNES, Humberto Hugo Nunes de ANDRADE, Danielle da Nóbrega ALVES, Gleycyelly Rodrigues ARAÚJO, Mirian Graciela da Silva Stiebbe SALVADORI, Reinaldo Nóbrega de ALMEIDA, Ricardo Dias de CASTRO

**Affiliations:** (a) Universidade Federal da Paraíba – UFPB, Faculty of Pharmacy, Department of Pharmaceutical Sciences, João Pessoa, PB, Brazil.; (b) Universidade Federal da Paraíba – UFPB, Faculty of Nursing, Department Development and Technological Innovation in Medicines, João Pessoa, PB, Brazil.; (c) Universidade Federal da Paraíba – UFPB, Faculty of Dentistry, Department of Clinical and Social Dentistry, João Pessoa, PB, Brazil.; (d) Universidade Federal da Paraíba – UFPB, Institute of Drugs and Medicines Research, Department of Psychology, João Pessoa, PB, Brazil.; (e) Universidade Federal da Paraíba – UFPB, Institute of Drugs and Medicines Research, Department of Physiology and Pathology, João Pessoa, PB, Brazil.

**Keywords:** Monoterpenes, Facial Pain, Oils, Volatile, Biological Products

## Abstract

This is a nonclinical, controlled, and triple-blind study to investigate the effects of codeine-associated geraniol on the modulation of orofacial nociception and its potential central nervous system depressing effect in an animal model. The orofacial antinociceptive activity of geraniol in combination with codeine was assessed through the following tests: (i) formalin-induced pain, (ii) glutamate-induced pain, and (iii) capsaicin-induced pain. Six animals were equally distributed into six groups and received the following treatments, given intraperitoneally (i.p.) 30 minutes before the experiments: a) geraniol/codeine 50/30 mg/kg; b) geraniol/codeine 50/15 mg/kg; c) geraniol/codeine 50/7.5 mg/kg; d) geraniol 50 mg/kg; e) codeine 30 mg/kg (positive control); or f) 0.9% sodium chloride (negative control). We performed pain behavior analysis after the injection of formalin (20 µL, 20%), glutamate (20 µL, 25 µM), and capsaicin (20 µL, 2.5 µg) into the paranasal region. Rubbing time of the paranasal region by the hind or front paw was used as a parameter. In the neurogenic phase of the formalin test, the geraniol/codeine at 50/7.5 mg/kg was able to promote the maximum antinociceptive effect, reducing nociception by 71.9% (p < 0.0001). In the inflammatory phase of the formalin test, geraniol/codeine at 50/30 mg/kg significantly reduced orofacial nociception (p < 0.005). In the glutamate test, geraniol/codeine at 50/30 mg/kg reduced the rubbing time by 54.2% and reduced nociception in the capsaicin test by 66.7% (p < 0.005). Geraniol alone or in combination does not promote nonspecific depressing effects on the central nervous system. Based on our findings, we suggest the possible synergy between geraniol and codeine in the modulation of orofacial pain.

## Introduction

Orofacial pain is a condition with either odontogenic or non-odontogenic etiology that affects soft and mineralized tissues (e.g., bones, skin, teeth, blood vessels, muscles, or glands) of the face and oral cavity. This type of pain can be difficult to assess and manage, and it has a direct impact on a person’s well-being.^1^ The committee of the Orofacial and Head Pain Special Interest Group of the International Association for the Study of Pain (IASP) considers that orofacial pain includes all the structures in the oral cavity and may be continuous, recurrent, or occasional. Besides, IASP uses codes for the different types of orofacial pain, including dentoalveolar disorders (pulpal, periodontal, gingival, oral mucosal, salivary gland, and jawbone pain), myofascial orofacial pain (primary and secondary, including acute and chronic pain), the temporomandibular joint (primary and secondary, including acute and chronic pain), lesion or disease of the cranial nerves (ex: trigeminal neuralgia), headaches, idiopathic orofacial pain, and psychosocial assessment of patients with orofacial pain. Considering all these problems, orofacial pain can affect 30% of the population.^2^

The treatment of orofacial pain includes the prescription of analgesic or anti-inflammatory drugs; however, adverse effects (e.g., gastrointestinal lesions, renal dysfunction, and pharmacological tolerance and dependence) limit the use of these drugs and cause treatment failure.^3^ The dependence-promoting effect of opioids, responsible for serious public health problems in developed countries, coupled with high rates of morbidity and mortality, highlights the need for prescriptions of lower doses of opioids or even their replacement with other pharmacological agents.^4^

Furthermore, while the opioid epidemic poses a serious problem, epidemiological data indicate that there is a significant portion of the population in need of strategies for the management of acute and chronic pain. Associating a low-potency opioid, such as codeine, with another drug to promote pain relief, either through modulation of nerve transmission or inflammatory response, represents an interesting therapeutic strategy. This allows for a reduction in the opioid analgesic dose while maintaining therapeutic efficacy. This indicates the need to develop new therapeutic approaches for the treatment of orofacial pain. Therefore, natural products have emerged as an alternative for the treatment of painful or inflammatory diseases.^5^

Geraniol (trans-3,7-dimethyl-2,6-octa-dien-1-ol), an acyclic monoterpene alcohol found in several essential oils obtained from aromatic plants, such as Cinnamomum tenuipilum and Valeriana officinalis. The pharmacological properties of this compound include antitumor, anti-inflammatory, antioxidative, and antimicrobial activities, in addition to hepatoprotective, cardioprotective, and neuroprotective effects.^6^

In the study of acute toxicity, the lethal dose 50% (LD_50_) of a substance is an important parameter to determine the relatively safe doses for conducting pharmacological studies.^7^La Rocca et al.^8^ evaluated the acute toxicity of geraniol through tests in which animals were treated with geraniol at 100, 300, and 1,000 mg/kg (p.o.) and 75, 100, 150, 200, and 300 mg/kg (i.p.). Our results indicate that the LD_50_ by the oral route is greater than 1,000 mg/kg and that geraniol has an estimated LD_50_ by the intraperitoneal route of 199.9 mg/kg.

Our previous studies have shown a probable inflammation-related antinociceptive effect of geraniol and reduced peripheral nerve excitability.^8^We found that geraniol was antinociceptive at doses of 25 mg/kg and 50 mg/kg (i.p.). The main predicted effect was mediated by the binding of geraniol to glutamatergic receptors (mGlur6, NMDA, and AMPA).^9^Considering the hypothesis that geraniol is associated with inflammatory response modulation and that it may additionally act on glutamatergic nociceptive receptors, its combination with codeine, which has a mechanism of action associated with opioid receptors, could represent an interesting therapeutic approach. This occurs because the drugs act on different protein targets, suggesting an additive effect.

These findings suggest that codeine-associated geraniol is a novel and effective therapy for the treatment of orofacial pain. This association allows for the reduction of the opioid dose and minimizes the relevant respiratory effects (e.g., tolerance, dependence, and respiratory depression). Therefore, this study aimed to investigate the effects of codeine-associated geraniol on modulating orofacial pain and its possible central nervous system depressing effect.

## Methods

### Experimental model

In this experimental, nonclinical, controlled, and triple-blind study, we evaluated healthy adult Swiss albino mice (Mus musculus) weighing 28 to 35 g for orofacial nociception. Mice were maintained under temperature-controlled conditions (21 ± 2°C) and a 12-hour light/dark cycle with food and water ad libitum. All animals were obtained from the Animal Facility “Prof. Dr. Thomas George”, Federal University of Paraiba, João Pessoa, State of Paraiba, Brazil.

The study protocol was approved by the Animal Research Ethics Committee of the Federal University of Paraíba (Protocol No. 3513111119/2021). Few mice were used in the experiments, and all measures were adopted to control their suffering or stress.

The sample was estimated using the difference in proportions and Cohen’s h effect size. The sample size (six animals per group) was calculated to provide a minimum power of 80% (1 – β) with an effect size (Hedge’s g) of 2.12 and a 95% confidence interval (one-tailed alpha error = 5%).^10^ Given the 20% attrition rate, the experimental groups consisted of six animals. The hypothesis that there was a difference in proportions between the two groups analyzed was tested.

### Tests for the induction of orofacial pain

We performed three tests to evaluate the orofacial antinociceptive activity of geraniol in combination with codeine: a) formalin-induced pain, b) glutamate-induced pain, and c) capsaicin-induced pain. We performed each test in three separate groups, and each group contained 36 animals divided into six subgroups of equally distributed mice. Animals were pretreated with codeine at 30 mg/kg (i.p.), geraniol at 50 mg/kg (i.p.), geraniol at 50 mg/kg (i.p.) and codeine at 30 mg/kg (i.p.), geraniol at 50 mg/kg (i.p.) and codeine at 15 mg/kg (i.p.), geraniol at 50 mg/kg (i.p.) and codeine at 7.5 mg/kg (i.p.), and saline (0.9% sodium chloride) followed by orofacial pain induction. Geraniol test dosages were determined based on the effects of geraniol on orofacial pain.^9^ All test substances were purchased from Sigma-Aldrich, City, USA.

The preparation, performance, and behavioral analysis were triple-blinded: researcher A classified the animals and prepared the materials; researcher B blindly administered the substances; researcher C performed the observation; researcher D performed the data analysis. Finally, researcher A reclassified the data analyzed by researcher D.

### Formalin-, glutamate-, and capsaicin-induced orofacial pain tests

The perinasal injection is justified by the specificity of the sensory innervation of the facial region of mice, which is performed by the trigeminal nerve (fifth cranial nerve). The trigeminal nerve fibers carry sensory stimuli of touch, pressure, temperature, and pain.^11^ Formalin was administered into the lateral portion of the upper lip (paranasal region) of mice half an hour after treatment using a 27-gauge needle with 20 µL of a 20% formalin solution (formaldehyde in distilled water). Mice were then observed for the next 40 minutes (neurogenic and inflammatory phases).

The glutamate-induced pain test was performed as described by Beirith et al.,^12^ with slight modifications. In this test, 20 µL of glutamate (25 µmol/L, Sigma-Aldrich, St. Louis, USA) was administered subcutaneously into the lateral portion of the right upper lip of mice with a 27-gauge needle half an hour after treatment. We then observed the animals for 15 minutes after the application of glutamate.

The capsaicin-induced pain test followed the protocol reported by Pelissier et al.^13^ and was adapted by Quitans-Júnior et al.^14^ This test mimics acute pain after a noxious stimulus and is useful for studying the mechanisms triggered by trigeminal nociception. It consisted in administering 20 μL of a 2.5 μg solution of capsaicin (Sigma Aldrich, Missouri, USA) into the right upper lip of mice half an hour after treatment. We observed nociceptive behavior for 20 minutes.

### General assessment of central nervous system - Rotarod test

The rotarod test is used to detect potential effects of muscle relaxation or lack of motor coordination induced by the administration of pharmacological agents, such as skeletal muscle relaxants or CNS depressants (e.g., anxiolytics). In this test, we place mice on a bar that rotates at a constant speed, allowing for the analysis of the animal’s ability to balance on it.^15^

One day before the test, the mice were pre-selected (without drug administration) according to the time spent on the rotating bar (7 rpm), which was set to three minutes in at least one of the three trials. On the test day, the sample was divided into six groups of six animals. Baseline measurement was performed. Subsequently, the mice were treated with codeine at 30 mg/kg (i.p.), geraniol at 50 mg/kg (i.p.), geraniol at 50 mg/kg plus codeine at 30 mg/kg (i.p.), geraniol at 50 mg/kg plus codeine at 15 mg/kg (i.p.), geraniol at 50 mg/kg plus codeine at 7.5 mg/kg (i.p.), diazepam at 4 mg/kg (i.p.), and the negative control group (0.9% sodium chloride). The mice were placed on the apparatus after half an hour of administration. This procedure was repeated at 60 and 120 minutes after drug administration.^16^

### Statistical analysis

The results obtained were analyzed using ANOVA followed by Tukey’s post-hoc test. The numerical data were entered into Graph Pad Prism version 7.0. The values obtained are expressed as mean ± standard deviation (SD). The significance level was set at 5% (p < 0.05).

## Results

### Formalin test

**Figure 1:**
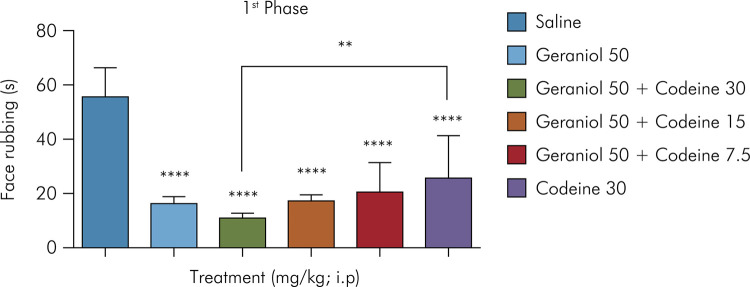
Effect of geraniol in combination with codeine on formalin-induced orofacial pain (neurogenic phase). Control (saline) and geraniol at 50 mg/kg (i.p.); geraniol and codeine (50 mg/kg plus 30 mg/kg, 50 mg/kg plus 15 mg/kg, and 50 mg/kg plus 7.5 mg/kg, respectively [i.p.]); codeine at 30 mg/kg (i.p.). Values are expressed as mean ± SD (six animals per group). *One-way ANOVA followed by Tukey’s post-hoc test was used for comparison between groups, **p < 0.01; ****p < 0.0001 relative to the negative control.

Administration of geraniol at 50 mg/kg plus codeine at 30 mg/kg resulted in a significant decrease in orofacial nociceptive behavior (p < 0.05) compared with the negative control in the formalin test in the neurogenic and inflammatory phases. The results shown in Figure 1 demonstrate a significant reduction of the orofacial rubbing time in animals treated with geraniol alone and also in animals treated with the combination of geraniol and codeine at geraniol doses of 50 mg/kg (12.5 ± 2.2), geraniol at 50 mg/kg plus codeine at 30 mg/kg (7.6 ± 1.7), geraniol at 50 mg/kg plus codeine at 15 mg/kg (11.25 ± 2.4), and geraniol at 50 mg/kg plus codeine at 7.5 mg/kg (15.6 ± 3.8), showing inhibition values of 77%, 86%, 79%, and 71.9%, respectively, compared with the negative control group (55.5 ± 4.47). Codeine at 30 mg/kg administered intraperitoneally decreased face-rubbing time by 52% (26.6 ± 5.25); however, in the neurogenic phase, the combination of geraniol at 50 mg/kg with codeine at 30 mg/kg was significantly more effective as compared to the group treated with the opioid alone at 30 mg/kg.

**Figure 2:**
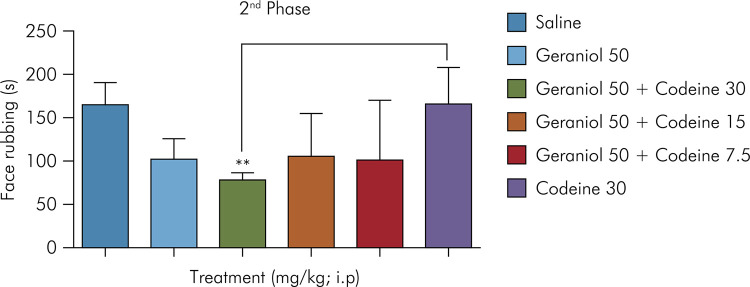
Effect of geraniol in combination with codeine on formalin-induced orofacial nociception (inflammatory phase). Control (saline) and geraniol at 50 mg/kg (i.p.); geraniol plus codeine (50 mg/kg plus 30 mg/kg, 50 mg/kg plus 15 mg/kg, and 50 mg/kg plus 7.5 mg/kg, respectively [i.p.]); codeine at 30 mg/kg (i.p.). Values are expressed as mean ± SD (six animals per group). One-way ANOVA followed by Tukey’s post-hoc test was used for comparison between groups, *p < 0.05; **p = 0.0026 vs. negative control.

Geraniol at 50 mg/kg in combination with codeine at 30 mg/kg also reduced the response time in the second phase of the formalin test (81 ± 2.8), with inhibition of nociception by 49.5% as compared to the negative control group (160.2 ± 12.02). The group treated with the standard drug (codeine at 30 mg/kg) showed no reduction in behavior (158.2 ± 17.57) (Figure 2).

### Glutamate test

**Figure 3:**
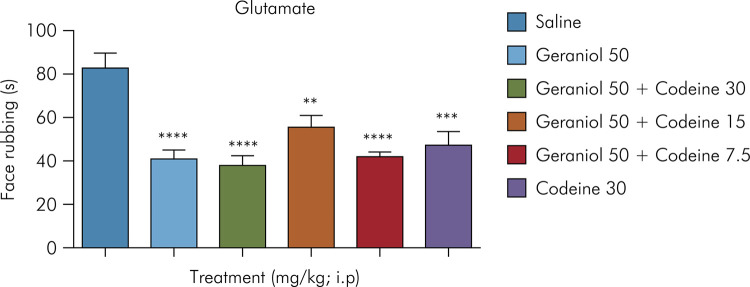
Effect of geraniol in combination with codeine on glutamate-induced orofacial nociception. Control (saline) and geraniol at 50 mg/kg (i.p.); geraniol and codeine (50 mg/kg plus 30 mg/kg, 50 mg/kg plus 15 mg/kg, and 50 mg/kg plus 7.5 mg/kg, respectively [i.p.]); codeine at 30 mg/kg (i.p.). Values are expressed as mean ± SD (six animals per group). *One-way ANOVA followed by Tukey’s post-hoc test was used for comparison between groups, **p = 0.0021; ***p < 0.001; ****p < 0001 compared with negative control.

According to Figure 3, geraniol alone and in combination with codeine were able to reduce the number of glutamate-induced face rubbing in mice at the following doses: geraniol at 50 mg/kg (40.67 ± 4), geraniol at 50 mg/kg plus codeine at 30 mg/kg (37.83 ± 4.4), geraniol at 50 mg/kg plus codeine at 15 mg/kg (55.67 ± 5.2), and geraniol at 50 mg/kg plus codeine at 7.5 mg/kg (42 ± 2.2), represented by the following percentages of orofacial nociception inhibition, respectively: 50.80%, 54.23%, 32.65%, and 49.19%, when compared to the negative control group (82.67 ± 6.6). As expected, codeine at 30 mg/kg administered intraperitoneally also inhibited orofacial nociception (46.95 ± 6.5).

### Capsaicin test

**Figure 4:**
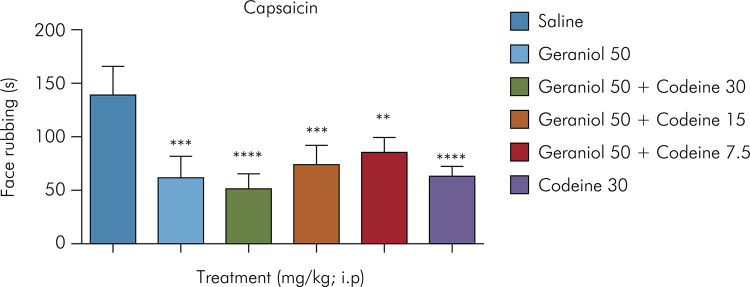
Effect of geraniol in combination with codeine on capsaicin-induced orofacial pain. Control (saline) and geraniol at 50 mg/kg (i.p.); geraniol and codeine (50 mg/kg plus 30 mg/kg, 50 mg/kg plus 15 mg/kg, and 50 mg/kg plus 7.5 mg/kg, respectively [i.p.]); codeine at 30 mg/kg (i.p.). Values are expressed as mean ± SD (six animals per group). *One-way ANOVA followed by Tukey’s post-hoc test was used for comparison between groups, **p = 0.001; ***p < 0.001; ****p < 0001 vs. negative control.

The results presented in Figure 4 show a significant reduction in face-rubbing time in animals treated with geraniol at 50 mg/kg plus codeine at 30 mg/kg (49.17 ± 13), geraniol at 50 mg/kg plus codeine at 15 mg/kg (74. 33 ± 14, 44), and geraniol at 50 mg/kg plus codeine at 7.5 mg/kg (86.5 ± 7.9), as well as those receiving the dose containing only geraniol at 50 mg/kg (71.8 ± 10.3), with the following orofacial nociception inhibition values, respectively: 66.7%, 49.67%, 41.43%, and 51.36%, as compared to the control group (147.7 ± 12.2). Codeine at 30 mg/kg administered intraperitoneally decreased face-rubbing time by 63.89% (53.3.8 ± 6.4).

### Rotarod test

**Figure 5:**
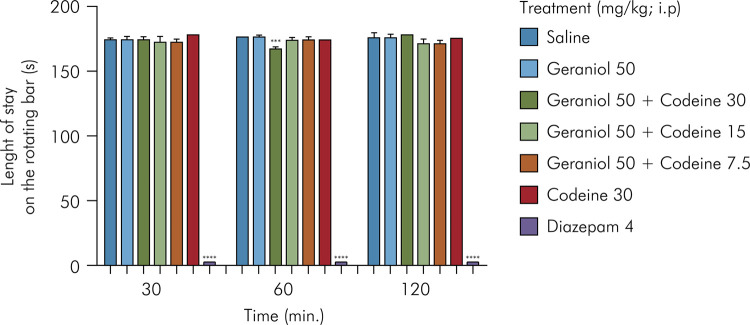
Effect of geraniol in combination with codeine on psychomotor function as assessed by the rotarod test. Control (saline) and geraniol (50 mg/kg, i.p.); geraniol and codeine (50 plus 30; 50 plus 15; 50 plus 7.5 mg/kg, i.p.); codeine (30 mg/kg, i.p.); diazepam (4 mg/kg, i.p.). Values are expressed as mean ± SD (six per group). *One-way ANOVA followed by Tukey’s post-hoc test was used for comparison between groups, ***p < 0.001, ****p < 0.0001 relative to the negative control.

The results presented in Figure 5 show no significant change in motor coordination in the rotarod test in animals treated with geraniol alone or in combination with codeine, except for the group of animals treated with geraniol at 50 mg/kg plus codeine at 30 mg/kg, which showed a reduction in time spent on the rotating bar at the 60-minute assessment. As expected, animals treated with diazepam at 4 mg/kg showed a significant reduction in time spent on the rotating bar at all assessment times.

## Discussion

The orofacial region is mainly innervated by A-delta and C fibers, which are responsible for transmitting nociceptive stimuli to the trigeminal nerve. The region is therefore susceptible to acute pain,^17^ and exposure to nociceptive substances (capsaicin, formalin, and glutamate) represents an important pharmacological investigative strategy for the discovery of new therapeutic approaches. In this study, we propose the association of geraniol with codeine as a strategy to promote pharmacological efficacy. Thus, we hope to help reduce the use of opioids, given their potential to promote chemical and psychological dependence and the alarming increase in morbidity and mortality associated with the misuse and abuse of prescription opioids.^4^ To circumvent this problem, we prescribed the use of geraniol at 50 mg/kg based on our previous data.^9^

The formalin test is a valid and reliable method to assess the processing and modulation of orofacial pain. Formalin is a chemical agent that directly and indirectly stimulates nociceptive fibers, thereby eliciting the nociceptive response^18^ and inducing the excitation of nociceptive neurons of the spinal trigeminal nucleus after application to the orofacial region. This test is divided into two phases: the neurogenic phase, which lasts from 0 to 5 minutes, and the inflammatory phase, which lasts from 15 to 40 minutes. In the neurogenic phase, there is a direct activation of nociceptors from C-type afferent fibers (i.e., a CNS-mediated pain sensation with the release of substance P and other neuropeptides). In the inflammatory phase, the interaction between the central and peripheral nervous systems is observed. Excitatory amino acids, nitric oxide, and peptides are released.^14,19^

We observed that the administration of codeine-associated geraniol promoted a decrease in formalin-induced orofacial nociceptive activity. The treated animals showed a reduction in face-rubbing time when compared to the control during the neurogenic phase. In this phase, animals treated with the combination of geraniol at 50 mg/kg with codeine at 30 mg/kg showed a greater reduction in face-rubbing time than animals treated with the standard drug alone, indicating a possible synergistic effect. In the neurogenic phase, all the combinations showed reduced orofacial pain scores, especially geraniol at 50 mg/kg combined with codeine at 7.5 mg/kg, as it was observed that the lower dose of opioid, when combined with geraniol, produced the maximum effect. This suggests the hypothesis that the association of geraniol at 50 mg/kg plus codeine at 7.5 mg/kg presents analgesic efficacy, with fewer undesirable effects caused by the opioid agent. Our findings indicate that geraniol, when combined with codeine, promotes a synergistic effect in the neurogenic phase of the formalin test. To better understand this effect, we suggest conducting new tests with variations in codeine doses based on isobologram analysis.^20^

In the inflammatory phase of the formalin test, only the animals treated with the combination of geraniol at 50 mg/kg and codeine at 30 mg/kg showed behavior indicative of pain reduction. This suggests that the combination of geraniol with codeine is beneficial because it expands the possibility of reducing neuronal excitation, also alleviating pain caused by the inflammatory process. As a weak opioid analgesic, codeine alone (30 mg/Kg) is not capable of modulating nociception induced by inflammatory mediators. Our findings suggest that the combination of codeine/geraniol is relevant, given that orofacial pains typically involve both nervous and inflammatory components. Previous results have reported the anti-inflammatory effect of geraniol,^21^ which can be explained by its participation in the regulation of N-methyl-D-aspartate (NMDA) receptors, the reduction of TNF-α levels,^22^ and the decrease of NO and PGE production in macrophages, probably by inhibition of iNOS and COX2.^23^ Other monoterpenes also showed similar behavior to geraniol in the two phases of the formalin test, such as citronellal,^14^ (R)-(+)-pulegone,^24^ (-)-linalool,^25^ and α, β-epoxy-carvone.^26^

The growing interest in investigations to determine the biological activity of monoterpenes as an option to treat pain and nociception has aroused because of the availability and favorable pharmacological qualities of these compounds as they can act on different pharmacological targets, reducing the possibility of the undesirable effects caused by drugs available for clinical use.^27^

Subcutaneous injection of glutamate causes intense excitation of nociceptors, resulting in a short-lived nociceptive response.^28^ This pattern involves peripheral, spinal, and supraspinal sites.^12^ This action occurs synergistically with the release of pro-inflammatory mediators and cytokines for nerve fiber excitation.^29^ In the present study, geraniol alone and in combination with codeine inhibited glutamate-induced orofacial pain, with better results when compared to the use of codeine alone. These findings suggest that geraniol interferes with the transmission of nociceptive information in the glutamatergic system, probably by interacting with N-methyl-D-aspartate (NMDA), kainate, and α-amino-3-hydroxy-5-methyl-4-isoxazolepropionic acid (AMPA) receptors.^9,12^

La Rocca et al.^8^ demonstrated the pain inhibitory potential of geraniol on glutamatergic pathways through pain induction experiments via intraplantar glutamate injection. Geraniol at 50 mg/kg via intraperitoneal injection caused a decrease in paw-licking time similar to the standard group pretreated with dizocilpine (MK-801), a nonselective NMDA receptor antagonist. Activation of glutamatergic receptors has also been shown to increase Ca^2^ ion influx, intracellular activation of metabolic pathways, and nitric oxide (NO) production and release.^19^ Considering that the effect of geraniol may be related to the suppression of NO and PDE production, probably by blocking iNOS and COX2,^24^ our findings suggest that geraniol exerts its effect by modulating glutamatergic receptors.

Capsaicin (8-methyl-N-vanilloyl-trans-6-zonisamide) is the main chemical component present in red pepper of the Capsicum genus and causes excitation of the nerve endings that transmit painful sensations.^30^ It activates transient receptors of the vanilloid family (TRPVs), especially TRPV1, which is one of the channels for Ca^2^ influx involved in cell migration, helping with pain transduction, mainly in the trigeminal pathway. The impulse transmission resulting from this activation is made by several mediators, including tachykinins, substance P, excitatory amino acids, NO, and other pro-inflammatory mediators.^31^

When pain was induced by capsaicin, all groups of treated animals showed a reduction in face-rubbing time when compared to the negative control. Geraniol alone and the combination at different doses reduced orofacial pain in mice. This effect suggests that geraniol may also modulate the TRPV1 receptor. Results of molecular docking studies through in silico evaluation predicted geraniol activity on TRPV1 receptors and receptors activated by excitatory peptides.^9^

By reducing the ability of the mice to walk, it is possible to assess whether the test substance promotes central nervous system depression. The rotarod test makes it possible to assess the integrity of the motor system, detecting neurological weakness and nonspecific muscle relaxation that could reduce motor coordination and invalidate the test results.^32^ According to the results obtained, the administration of geraniol alone or in combination with codeine did not affect motor coordination in mice at 30 and 120 minutes. However, at 60 minutes and at the geraniol dose of 50 mg/kg combined with codeine at 30 mg/kg, a small change in the time spent on the rotating bar was observed when compared to the negative control group. However, it is important to emphasize that this possible depression observed at 60 minutes was transient because it did not appear at 120 minutes, and it was small compared to the depressing effect caused by diazepam.

Our findings suggest that codeine-associated geraniol has orofacial antinociceptive activity and may be an advantageous alternative because of the broader spectrum of action when compared to the effect produced by codeine and geraniol alone on different signal transduction pathways. This study suggests that future investigations should consider the hypothesis that geraniol acts on mGlur6, NMDA, AMPA, μ, ĸ, and δ opioid receptors, and TRPV1. Elucidation of the mechanism of action is critical for understanding the pharmacological effect and potential clinical application.

## References

[1] Matsuka Y (2022). Orofacial pain: molecular mechanisms, diagnosis, and treatment 2021. Int J Mol Sci.

[2] ICOP, International classification of orofacial pain (2020). Cephalalgia.

[3] Bindu S, Mazumder S, Bandyopadhyay U (2020). Non-steroidal anti-inflammatory drugs (NSAIDs) and organ damage: a current perspective. Biochem Pharmacol.

[4] Upp LA, Waljee JF (2020). The opioid epidemic. Clin Plast Surg.

[5] Newman DJ, Cragg GM (2020). Natural products as sources of new drugs over the nearly four decades from 01/1981 to 09/2019. J Nat Prod.

[6] Lei Y, Fu P, Jun X, Cheng P (2019). Pharmacological properties of geraniol-a review. Planta Med.

[7] Sá RCS, Andrade LN, Sousa DP (2013). A review on anti-inflammatory activity of monoterpenes. Molecules.

[8] La Rocca V, Fonsêca DV, Silva-Alves KS, Ferreira-da-Silva FW, Sousa DP, Santos PL (2017). Geraniol induces antinociceptive effect in mice evaluated in behavioural and electrophysiological models. Basic Clin Pharmacol Toxicol.

[9] Costa TK, Barros MS, Braga RM, Viana JO, Sousa FB, Scotti L (2020). Orofacial antinociceptive activity and anchorage molecular mechanism in silico of geraniol. Braz Oral Res.

[10] Cohen J (1998). Statistical power analysis for the behavioral sciences.

[11] Chichorro JG, Porreca F, Sessle B (2017). Mechanisms of craniofacial pain. Cephalalgia.

[12] Beirith A, Santos AR, Calixto JB (2002). Mechanisms underlying the nociception and paw oedema caused by injection of glutamate into the mouse paw. Brain Res.

[13] Pelissier T, Pajot J, Dallel R (2002). The orofacial capsaicin test in rats: effects of different capsaicin concentrations and morphine. Pain.

[14] Quintans LJ, Melo MS, Sousa DP, Araújo AA, Onofre AC, Gelain DP (2010). Antinociceptive effects of citronellal in formalin-, capsaicin-, and glutamate-induced orofacial nociception in rodents and its action on nerve excitability. J Orofac Pain.

[15] Dunham NW, Miya TS, Edwards LD (1957). The pharmacological activity of a series of basic esters of mono- and dialkylmalonic acids. J Am Pharm Assoc.

[16] Mendes FR, Mattei R, Carlini ELA (2002). Activity of Hypericum brasiliense and Hypericum cordatum on the central nervous system in rodents. Fitoterapia.

[17] Raboisson P, Dallel R (2004). The orofacial formalin test. Neurosci Biobehav Rev.

[18] Luccarini P, Childeric A, Gaydier AM, Voisin D, Dallel R (2006). The orofacial formalin test in the mouse: a behavioral model for studying physiology and modulation of trigeminal nociception. J Pain.

[19] Silva JC, Macedo LA, Souza GR, Oliveira-Junior RG, Lima-Saraiva SR, Lavor ÉM (2016). Orofacial antinociceptive effect of the ethanolic extract of Annona vepretorum Mart. (Annonaceae). Z Naturforsch C J Biosci.

[20] Huang RY, Pei L, Liu Q, Chen S, Dou H, Shu G (2019). Isobologram analysis: a comprehensive review of methodology and current research. Front Pharmacol.

[21] Tiwari M, Kakkar P (2009). Plant derived antioxidants - Geraniol and camphene protect rat alveolar macrophages against t-BHP induced oxidative stress. Toxicol In Vitro.

[22] Lv Y, Zhang L, Li N, Mai N, Zhang Y, Pan S (2017). Geraniol promotes functional recovery and attenuates neuropathic pain in rats with spinal cord injury. Can J Physiol Pharmacol.

[23] Su YW, Chao SH, Lee MH, Ou TY, Tsai YC (2010). Inhibitory effects of citronellol and geraniol on nitric oxide and prostaglandin E ^2^ production in macrophages. Planta Med.

[24] Sousa DP, Nóbrega FF, Lima MR, Almeida RN (2011). Pharmacological activity of (R)-(+)-pulegone, a chemical constituent of essential oils. Z Naturforsch C J Biosci.

[25] Venâncio AM, Marchioro M, Estavam CS, Melo MS, Santana MT, Onofre AS (2011). Ocimum basilicum leaf essential oil and (-)-linalool reduce orofacial nociception in rodents: a behavioral and electrophysiological approach. Rev Bras Farmacogn.

[26] Rocha ML, Oliveira LE, Santos CCP, Sousa DP, Almeida RN, Araújo DA (2013). Antinociceptive and anti-inflammatory effects of the monoterpene a,ß-epoxy-carvone in mice. J Nat Med.

[27] Guimarães AG, Xavier MA, Santana MT, Camargo EA, Santos CA, Brito FA (2012). Carvacrol attenuates mechanical hypernociception and inflammatory response. Naunyn Schmiedebergs Arch Pharmacol.

[28] Turner SP, Outhwaite W (2007). The SAGE handbook of social science methodology.

[29] Bernardino L, Xapelli S, Silva AP, Jakobsen B, Poulsen FR, Oliveira CR (2005). Modulator effects of interleukin-1ß and tumor necrosis factor-a on AMPA-induced excitotoxicity in mouse organotypic hippocampal slice cultures. J Neurosci.

[30] Caterina MJ, Schumacher MA, Tominaga M, Rosen TA, Levine JD, Julius D (1997). The capsaicin receptor: a heat-activated ion channel in the pain pathway. Nature.

[31] Waning J, Vriens J, Owsianik G, Stüwe L, Mally S, Fabian A (2007). A novel function of capsaicin-sensitive TRPV1 channels: involvement in cell migration. Cell Calcium.

[32] Souza MM, Pereira MA, Ardenghi JV, Mora TC, Bresciani LF, Yunes RA (2009). Filicene obtained from Adiantum cuneatum interacts with the cholinergic, dopaminergic, glutamatergic, GABAergic, and tachykinergic systems to exert antinociceptive effect in mice. Pharmacol Biochem Behav.

